# AI in Psychiatry: Obstacles and Opportunities

**DOI:** 10.1192/bjo.2025.10245

**Published:** 2025-06-20

**Authors:** Adam Whyte, Kilda Carpenter

**Affiliations:** Oxford Health NHS Foundation Trust, Oxford, United Kingdom

## Abstract

**Aims:** This study aims to evaluate the attitudes of resident doctors toward the use of artificial intelligence (AI) in clinical psychiatric practice. As AI technology continues to advance, its potential to support patient care is becoming increasingly significant, and understanding the perspectives of resident doctors provides crucial insights into how AI can be integrated effectively into psychiatric practice.

**Methods:** An anonymised online survey was sent to resident and speciality doctors of different grades to elicit their views on the use of AI in psychiatric practice. The survey consisted of Likert scale and multiple-choice questions relating to attitudes towards AI and perceived barriers to its clinical use. Participants were also invited to share their thoughts in a free-text question at the end of the survey.

**Results:** 41 resident doctors replied to the survey. 83% of responders had used AI personally, 78% of whom had used ChatGPT. There was clear consensus that AI could help with written tasks, with 85% of doctors reporting that they felt AI could help with summarising notes, and 78% indicating that it could support writing clinical letters. Only 34% of responders felt that AI could help with more complex generative tasks, such as guiding risk assessment, suggesting treatment options or interpreting investigation results.

There were evident barriers to the adoption of AI with lack of trust in the accuracy/reliability of the information produced (82%) and concerns about possible bias in the algorithms/data used by AI (84%). Legal (71%) and ethical concerns (67%) were also widely reported as barriers to its adoption.

Attitudes to using AI initially varied, with 29% of doctors reporting that they were “very unlikely” or “unlikely” to use AI in their clinical practice. After engaging with the concepts presented in the survey, attitudes demonstratably shifted with this figure dropping to 20%.

**Conclusion:** This study demonstrates the diversity of opinion surrounding the use of AI among resident doctors in psychiatry. The majority of respondents agreed that AI would be of use in administrative tasks, but felt that the main barriers to its use included concerns about the accuracy of the generated information, and ethicolegal considerations, particularly related to information governance.

